# Applying causal models to explore the mechanism of action of simvastatin in progressive multiple sclerosis

**DOI:** 10.1073/pnas.1818978116

**Published:** 2019-05-09

**Authors:** Arman Eshaghi, Rogier A. Kievit, Ferran Prados, Carole H. Sudre, Jennifer Nicholas, M. Jorge Cardoso, Dennis Chan, Richard Nicholas, Sebastien Ourselin, John Greenwood, Alan J. Thompson, Daniel C. Alexander, Frederik Barkhof, Jeremy Chataway, Olga Ciccarelli

**Affiliations:** ^a^Queen Square Multiple Sclerosis Centre, Department of Neuroinflammation, UCL Queen Square Institute of Neurology, Faculty of Brain Sciences, University College London, London WC1B 5EH, United Kingdom;; ^b^Centre for Medical Image Computing, Department of Computer Science, University College London, London WC1E 6BT, United Kingdom;; ^c^Max Planck University College London Centre for Computational Psychiatry and Ageing Research, London WC1B 5EH, United Kingdom;; ^d^MRC Cognition and Brain Sciences Unit, University of Cambridge, Cambridge CB2 7EF, United Kingdom;; ^e^Centre for Medical Image Computing, UCL Department of Medical Physics and Biomedical Engineering, University College London, London WC1E 6BT, United Kingdom;; ^f^Universitat Oberta de Catalunya, Barcelona 08018, Spain;; ^g^School of Biomedical Engineering and Imaging Sciences, King’s College London, London WC2R 2LS, United Kingdom;; ^h^Dementia Research Centre, UCL Queen Square Institute of Neurology, University College London, London WC1N 3AR, United Kingdom;; ^i^UCL Department of Medical Physics and Biomedical Engineering, University College London, London WC1E 6BT, United Kingdom;; ^j^London School of Hygiene and Tropical Medicine, London WC1E 7HT, United Kingdom;; ^k^Department of Clinical Neurosciences, University of Cambridge, Cambridge CB2 0QQ, United Kingdom;; ^l^Division of Brain Sciences, Imperial College London, London W12 0NN, United Kingdom;; ^m^University College London Institute of Ophthalmology, University College London, London EC1V 9EL, United Kingdom;; ^n^National Institute for Health Research, University College London Hospitals Biomedical Research Centre, London W1T 7DN, United Kingdom;; ^o^Department of Brain Repair and Rehabilitation, UCL Queen Square Institute of Neurology, University College London, London WC1B 5EH, United Kingdom;; ^p^Department of Radiology and Nuclear Medicine, Vrije Universiteit Medisch Centrum, 1007 MB Amsterdam, The Netherlands

**Keywords:** causal modeling, multiple sclerosis, clinical trial, structural equation modeling, progressive MS

## Abstract

Understanding the mode of action of drugs is a challenge with conventional methods in clinical trials. Here, we aimed to explore whether simvastatin effects on brain atrophy and disability in secondary progressive multiple sclerosis (SPMS) are mediated by reducing cholesterol or are independent of cholesterol. We applied structural equation models to the MS-STAT trial in which 140 patients with SPMS were randomized to receive placebo or simvastatin. At baseline, after 1 and 2 years, patients underwent brain magnetic resonance imaging; their cognitive and physical disability were assessed on the block design test and Expanded Disability Status Scale (EDSS), and serum total cholesterol levels were measured. We calculated the percentage brain volume change (brain atrophy). We compared two models to select the most likely one: a cholesterol-dependent model with a cholesterol-independent model. The cholesterol-independent model was the most likely option. When we deconstructed the total treatment effect into indirect effects, which were mediated by brain atrophy, and direct effects, simvastatin had a direct effect (independent of serum cholesterol) on both the EDSS, which explained 69% of the overall treatment effect on EDSS, and brain atrophy, which, in turn, was responsible for 31% of the total treatment effect on EDSS [β = −0.037; 95% credible interval (CI) = −0.075, −0.010]. This suggests that simvastatin’s beneficial effects in MS are independent of its effect on lowering peripheral cholesterol levels, implicating a role for upstream intermediate metabolites of the cholesterol synthesis pathway. Importantly, it demonstrates that computational models can elucidate the causal architecture underlying treatment effects in clinical trials of progressive MS.

## Significance

Traditional analysis of clinical trials precludes a mechanistic understanding of drug actions. This is further compounded by the use of outcome measures in clinical trials not directly related to the mechanism of action of the medication under study. Here, we applied structural equation models to the double-blind randomized controlled trial of simvastatin in secondary progressive multiple sclerosis to investigate causal associations that underlie treatment effects. Our results suggest that beneficial effects of simvastatin on reducing the rate of brain atrophy and slowing the deterioration of disability are independent of serum cholesterol reduction. Our work demonstrates that structural models can elucidate the statistical pathways underlying treatment effects in clinical trials of poorly understood neurodegenerative disorders, such as progressive multiple sclerosis.

Understanding mechanisms underpinning progression in multiple sclerosis (MS) is a significant challenge and a major research focus (1). Therefore, the mode of action of potential therapies for progressive MS is difficult to elicit. This is further compounded by the use of outcome measures in clinical trials that may not relate directly to the mechanism of action of the medication under study (1). The challenge of understanding the mode of action of a medication is exemplified by the simvastatin trial, a phase 2 trial for secondary progressive MS (2), in which MRI measures of atrophy and clinical disability showed beneficial effects. The fundamental question as to whether simvastatin’s beneficial effects on clinical outcomes and brain atrophy were mediated by lowering peripheral cholesterol levels was impossible to answer (3).

Mechanistic computational methods can elucidate the most plausible chain of events, by simultaneous analysis of multimodal data; these models assess hypothesized causal (and statistical) associations linking intermediate variables to outcomes of interest (4). They have been employed in clinical trials of Alzheimer’s disease (5), neurocognitive aging (6), and more extensively in social sciences (7). Applying multivariate mechanistic models to the simvastatin trial allows a quantitative comparison of the statistical pathways resulting in the observed effects of simvastatin on clinical outcomes clarifying the mechanisms underpinning its effect. An improved understanding of these statistical pathways will show that this methodology can be extended to other trials to obtain insights into the mechanisms through which experimental therapies provide clinical benefit.

In this study, we reanalyzed the MS-STAT trial data and modeled hypothesized causal associations by which simvastatin leads to changes in brain atrophy, clinical and cognitive outcome measures, either directly or indirectly via changes in peripheral cholesterol level. We tested the hypothesis that the reduction in serum cholesterol levels mediated the impact of simvastatin on brain atrophy and on disability against the alternative hypothesis that simvastatin effects were independent of peripheral cholesterol level. A subsidiary aim was to investigate whether the effect of simvastatin on brain atrophy was targeting specific regions.

## Materials and Methods

### Participants.

This was a post hoc study that included participants of the MS-STAT trial (ClinicalTrials.gov registration number: NCT00647348) (2). MS-STAT was a phase 2 double-blind randomized controlled trial whose primary and preplanned analyses have been reported previously (2, 8). Briefly, the eligibility criteria were as follows: (*i*) age between 18 and 65 y, (*ii*) Expanded Disability Status Scale (EDSS) (9) of between 4.0 and 6.5, (*iii*) fulfilling revised 2005 McDonald criteria (10), and (*iv*) secondary progressive MS defined by clinically confirmed disability worsening over the preceding 2 y. Detailed eligibility criteria are available elsewhere (2). The trial protocol was reviewed and approved by the Institutional Review Board at each study center (Charing Cross Hospital, The Chalfont Centre, Buckinghamshire, UK, and Hurstwood Park Hospital, Surrey, UK). Ethics was granted by Berkshire Research Ethics Committee (reference 07/Q1602/73). All participants gave informed consent before entering this study.

### Imaging Protocol.

Patients were scanned at each visit (three visits in total) with 3D T1-weighted, double-echo proton density (PD) and T2-weighted MRI at two imaging centers in the United Kingdom with 1.5- and 3-T scanners. The same scanner and imaging protocol were used for an individual participant throughout the trial. “Scanner” was a minimization variable (as explained above) between treatment and placebo groups. Acquisition protocols are reported elsewhere (2).

### Clinical and Cognitive Outcomes.

Patients underwent comprehensive clinical and cognitive assessments. Here, we studied those outcomes that had shown significant (or marginally significant) changes in previous reports (2, 8), which were the following: the total cholesterol level, EDSS, Multiple Sclerosis Impact Scale-29v2 (MSIS-29v2) (total score and physical subscale) (11), Wechsler Abbreviated Test of Intelligence (WASI) block design test (T score) (12), paced-auditory serial addition test (PASAT) (13), and Frontal Assessment Battery (FAB) (14).

### Image Analysis.

We performed image analysis based on our established pipeline for patients with MS, which is similar to what we have previously reported (15). Our goals were to extract regional volumes, T2 lesion masks, and the whole-brain percentage volume change with SIENA (16). Briefly, the pipeline included N4-bias field correction of T1-weighted scans to reduce intensity inhomogeneity (17), constructing a symmetric within-subject template for unbiased atrophy calculation (18), rigid transformation of T1, PD, and T2 sequences to the within-subject unbiased symmetric space, automatic longitudinal lesion segmentation of visible T2 lesions with Bayesian model selection (19, 20), manual editing of these lesion masks and quality assurance with the 3D-Slicer software, filling of hypointense lesions in T1 scans (21), and brain segmentation and parcellation with geodesic information flows (GIF) software (22). Technical details are given in *SI Appendix*, *Supplemental Methods*. Outputs of this pipeline were the following: (*i*) percentage whole-brain volume change (SIENA PBVC), (*ii*) T2 lesion masks, and (*iii*) regional brain volumes according to Neuromorphometrics’ atlas, which is similar to the Desikan–Killiany–Tourville (23) atlas available at https://scalablebrainatlas.incf.org/human/NMM1103; for each region, we summed volumes of the left and right hemispheres.

### Statistical Analysis.

We employed separate mixed-effects models to calculate the differences in the rate of changes in brain volume loss, EDSS, and cognitive scores (PASAT, frontal assessment battery, and block design T score) over time between the two arms of the trial. The aim of these analyses, which are different from the statistical tests carried out in the previous publication of this trial (2, 8), was to identify variables that showed a significant difference in their rates of change between treated and placebo arms and can be entered in the subsequent multivariate analysis (see below). Demographic and disease characteristics and the details of these mixed-effect models and the corresponding results are given in *SI Appendix*.

### Multivariate Analysis.

We performed multivariate analyses in the following steps:

*i*) Variable selection using the above (mixed-effects) univariate analyses: to limit the analysis to measures with significant rates of change.
*ii*) Model construction: to formulate mechanistic hypotheses as structured statistical models.
*iii*) Model selection: to choose the most likely hypothesis.
*iv*) Parameter estimation: to quantify, in the most likely model, pathways between serum cholesterol levels, brain atrophy, cognitive, patient-reported outcome measure, and clinical variables.

### Variable Selection and Model Construction.

We implemented multivariate analysis with structural equation modeling. Specifically, we fit a series of path models, which test whether a set of causal relationships is compatible with the observed associations. We used Lavaan package, version 0.5–23 (24), in R. Structural equation models allow simultaneous fitting of several regression models to quantify statistical pathways between variables. We included outcomes from the univariate analyses (explained above) that had significant differences in their rate of change between placebo and simvastatin groups. Since nuisance variables (age, gender, and center) did not affect the above univariate analyses, we did not include them in multivariate models. We only entered the physical subtest of MSIS-29v2 (instead of the total score) in structural equation models, because changes in this subtest drove the change in total score. Similarly, we entered the block design test because the mixed-effects models showed a significant difference in this test between the treated and the placebo arm (*SI Appendix*). We calculated the difference between baseline and second-year values for each variable and divided it by 2. We refer to this as the “annualized change” throughout this manuscript.

**Fig. 1.**
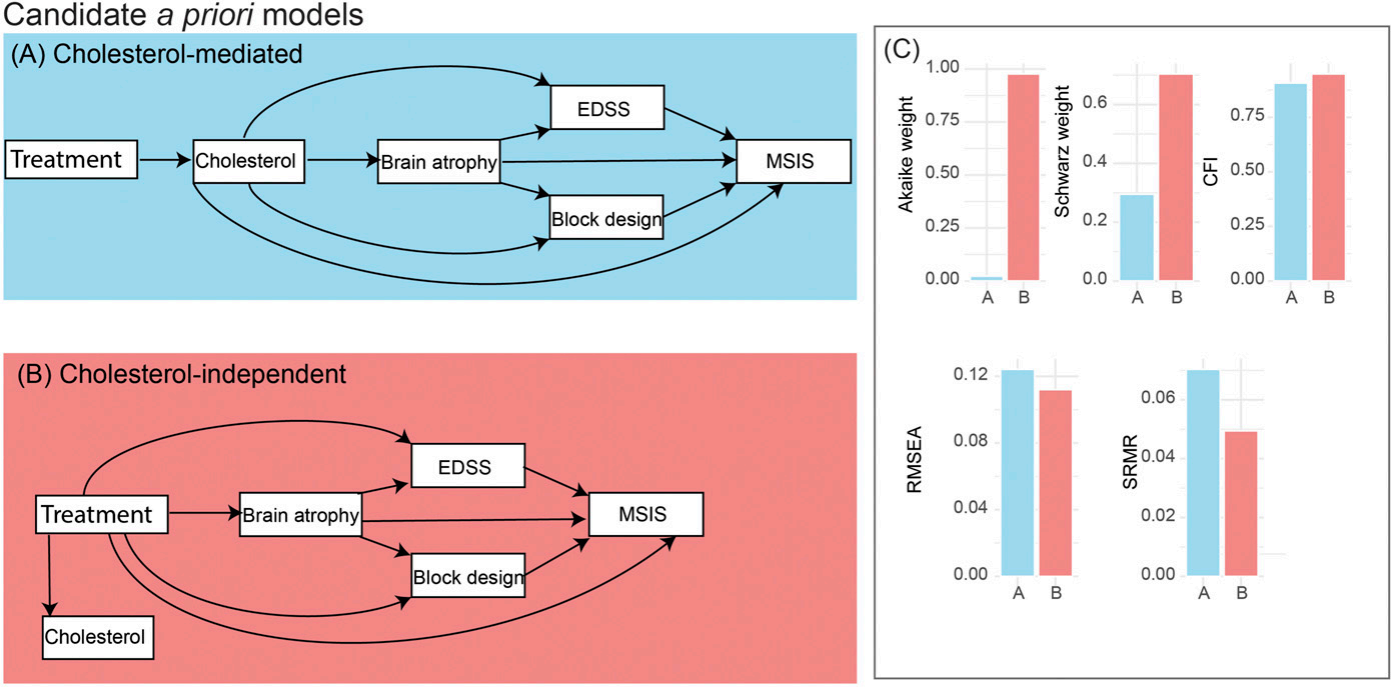
Model *A* or cholesterol-mediated model assumes that the cholesterol-lowering effect of simvastatin is the cause of the slowing of the brain atrophy and disability worsening. Model *B* or cholesterol-independent (or pleiotropic) model assumes that the cholesterol-lowering effect of simvastatin is independent of its effect on brain atrophy and clinical outcomes. In both models, a lower rate of brain atrophy development has an effect on the clinical change, as measured by the EDSS, block design, and MSIS-29v2. Additionally, in both models, the physical subscore of MSIS-29v2 (that showed significant effect of treatment) is included as the last variable in the cascade of events, because it is a subjective patient-reported outcome measure. All of the variables are “annualized” and represent annual rates of change between baseline and second-year follow-up visits. Each rectangle represents a variable. The arrows represent multivariate regressions, where an arrow starts from a predictor and points to the dependent variable. *C* compares fit-measures that are shown on the *y* axis of each of the five bar plots with models *A* and *B* on the *x* axis. Blue corresponds to cholesterol-mediated model, and red, to cholesterol-independent model. Fit measures suggest that cholesterol-independent model (or model *B*) was the most likely model given data, because it had a higher Akaike and Schwarz weights, higher CFI, lower SRMR, and lower RMSEA. CFI, confirmatory factor index; EDSS, Expanded Disability Status Scale; MSIS, Multiple Sclerosis Impact Scale; PBVC, percentage brain volume change; RMSEA, root-mean-squared error of approximation; SRMR, standardized root-mean-square residual.

We hypothesized two a priori models to explain relationships between these variables according to the literature (25, 26) and on the basis of our opinion. The first is a cholesterol-mediated model, in which the effects of simvastatin on clinical measures (both physical and cognitive) and brain atrophy are mediated by changes in cholesterol (Fig. 1*A*). The second is a cholesterol-independent model, in which simvastatin has a direct effect on the clinical and MRI outcome measures, independent of its effect on serum cholesterol levels (Fig. 1*B*). In both models, the rate of brain atrophy development has a direct effect on clinical change, as measured by the EDSS, block design, and MSIS-29v2 (Fig. 1). In both models, we included MSIS-29v2 (physical subscore) as the last variable in the cascade of events, because it is a subjective patient-reported questionnaire of physical ability expected to reflect the impact that clinical and cognitive impairment has on patient’s quality of life.

### Model Selection and Parameter Estimation.

We fitted both the cholesterol-mediated and cholesterol-independent model (shown in Fig. 1) using full-information maximum likelihood to adjust for missingness, and with the robust SEs to account for nonnormality (e.g., EDSS). We assessed the goodness of fit for each model and reported the parameters for the most likely model. To evaluate overall fit of a model, we used the comparative fit index (CFI) (compares the fit of the model with a model with uncorrelated variables; acceptable fit > 0.95; good fit > 0.97), standardized root-mean-square residual (square root of the average of the covariance of residuals; good fit < 0.08), and root-mean-squared error of approximation (RMSEA) (discrepancy between the model and population covariance; good fit < 0.06) (27). To estimate the relative quality of a model given the data, we calculated information criteria [Akaike information criterion (AIC) and Bayesian information criterion (BIC)] of each model. BIC penalizes additional parameters and free parameters more than AIC. BIC assumes that the true model is among the candidate models, while AIC assumes that the true model is unknown. We used different model comparison measures and several goodness-of-fit measures to make sure that our results were confirmed when using different methods. Since raw AIC and BIC values do not have a meaningful scale, we calculated the Akaike and Schwarz weights to represent the conditional probability of each model given the data directly (28). To have an unbiased estimate, we calculated fit measures (mentioned above) iteratively on 1,000 bootstrap samples and reported the median of bootstrap results with 95% confidence intervals.

### Bayesian Mediation Models.

To calculate how much of the total treatment effect was mediated by intermediate variables, we constructed post hoc models for variables involved in the significant pathways of a priori models (explained above). Each model included three variables: treatment, an intermediate variable, and a final outcome. Intermediate and outcome variables were the rates of annual change of the following variables: total cholesterol level, brain atrophy, EDSS, and block design score. Here, we used Bayesian multivariate models to report credible intervals (CIs), especially for those of cholesterol-mediated pathways, instead of *P* values and confidence intervals to allow an easier interpretation of nonsignificant findings. This enabled testing whether the lack of statistically significant cholesterol-mediated effects were because of lack of statistical power or there was evidence for the absence of cholesterol-mediation effects of simvastatin (29, 30). We used Blavaan package, version 0.3–2.283 (31), inside R, version 3.4.0 (32). In the Bayesian analysis, we considered an effect to be significant when the 95% CI of a parameter did not cross zero. We discarded the first 4,000 (“burn-in” samples) and reported the next 10,000 samples as posterior distributions with Markov chain Monte Carlo method with Stan, version 2.16.0 (33). We used noninformative uniform priors for Bayesian analyses.

### Regional Brain Atrophy Analysis.

To investigate whether the effect of simvastatin was predominant in, and limited to, certain brain regions, we carried out univariate mixed-effects models to compare regional atrophy rates between trial arms, by adjusting for age, gender, center, and total intracranial volume (34).We summed respective regions from left and right hemispheres and constructed linear mixed-effects models for each area (∼60 models), where the volume of a given area was the dependent variable. Independent variables (fixed effects and random effects) were similar to the models used for cognitive and clinical outcomes with an additional variable for total intracranial volume to adjust for the head size (34) and scanner (1.5 or 3 T). First, we extracted rates of atrophy for those regions that showed a significant rate of change (significant slope, *P* < 0.05), after adjustment for multiple comparisons with the false-discovery rate (35). With a similar model, we calculated the rate of change within the treatment and placebo groups. Therefore, we reported brain regions that showed a significant rate of change in the combined treatment and placebo groups as well as separate rates within each group. To explore whether the effect of simvastatin on EDSS was mediated by regional atrophy, we performed mediation analysis with the following variables:

*i*) Predictor variable: treatment (categorical: simvastatin or placebo);
*ii*) Mediator variable: volume change in the area with the largest effect of treatment (transverse temporal gyrus);
*iii*) Dependent variable: EDSS.

For regional mediation analysis, we employed the same methodology as explained above (*Multivariate Analysis*).

We also performed a focused analysis on the volume of medulla oblongata (to capture spinal cord related pathology in the absence of spinal cord imaging data), which is explained in *SI Appendix*.

### Code and Material Availability.

Computer codes with simulated data for this manuscript can be found at https://github.com/armaneshaghi/causalTrialModel. The ethical approval of this project restricts public release of the raw dataset.

## Results

### Multivariate Analysis: Simvastatin Effect on Clinical Outcomes and Brain Atrophy Is Independent of Cholesterol.

**Fig. 2.**
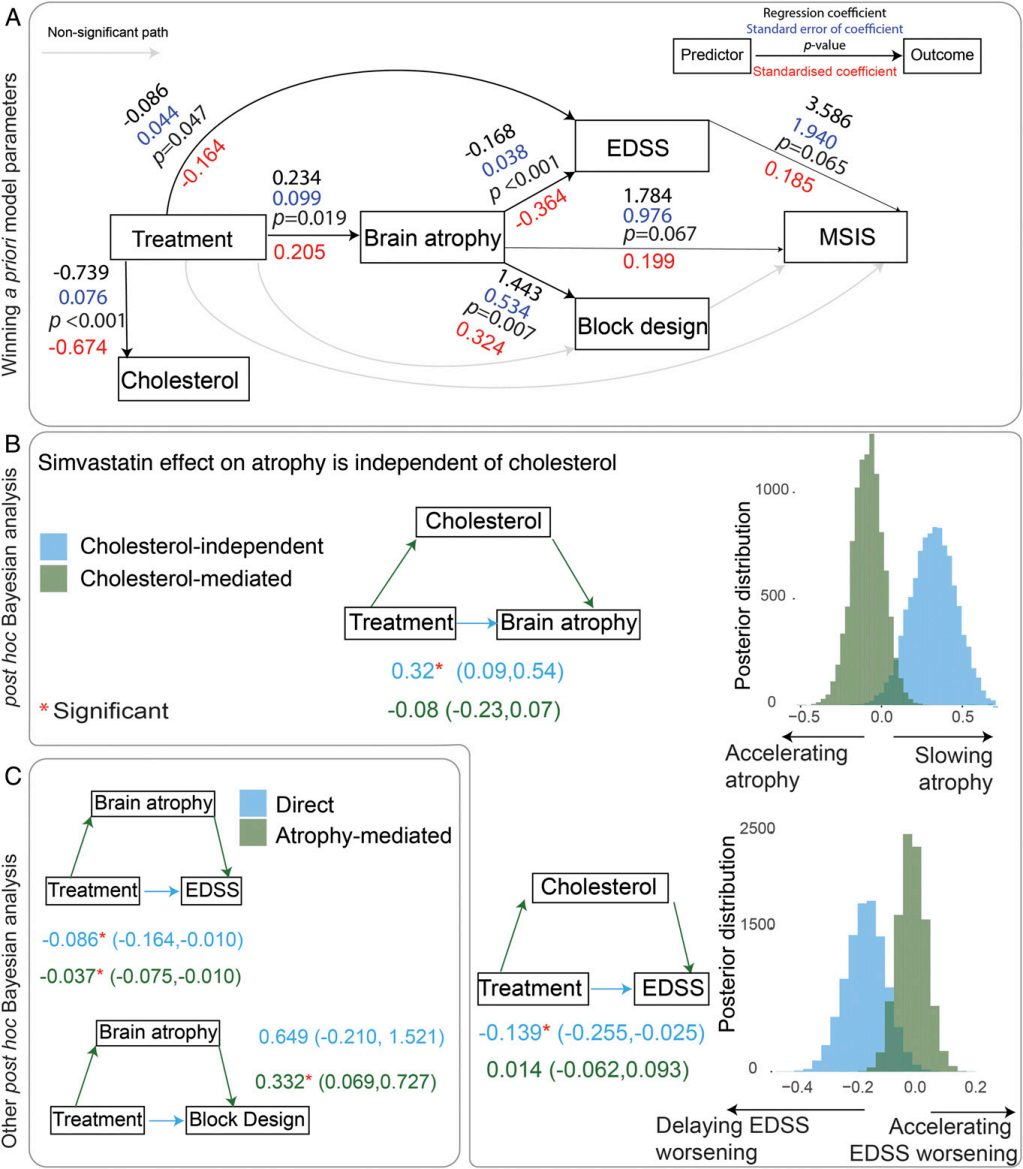
*A* shows the parameter estimates of the winning model, which is model *B* in Fig. 1. Each arrow is a regression “path,” where the arrow starts from the predictor(s) and points to the dependent variable(s). Significant paths (*P* < 0.05) are shown with bold arrows, while nonsignificant paths are thinner. The black numbers on each arrow represent regression coefficients and their *P* values. The blue numbers represent SEs of the coefficients. The red numbers represent standardized coefficients. *B* shows the Bayesian post hoc analysis of cholesterol-mediated pathway vs. direct pathway that does not depend on cholesterol to slow brain atrophy. The results confirm that a direct pathway (cholesterol-independent) slows brain atrophy. The numbers on the *Left* side of the *B* show median of the posterior distribution of the model parameters, and the numbers inside parentheses show 95% credible intervals (CIs). The 95% CIs of coefficients of direct pathway and cholesterol-mediated pathways do not overlap; this suggests that the lack of significance in cholesterol-mediated pathway is unlikely to be due to a lack of statistical power. We used a Bayesian method to ease the interpretation of nonsignificant findings and to report CIs (rather than the confidence intervals). *B* also shows Bayesian mediation analyses for brain atrophy and EDSS. The direct effect is shown in blue and the mediation effect (or indirect effect) is shown in green. The treatment effect on brain atrophy is independent of its effect on cholesterol because the 95% CIs do not overlap. Brain atrophy mediates 31% of the treatment effect on EDSS. *C* shows mediation analysis for other variables. They can be interpreted similarly. EDSS, Expanded Disability Status Scale; MSIS, Multiple Sclerosis Impact Scale (physical subtest); PBVC, percentage brain volume change.

The cholesterol-independent model, in which simvastatin has a direct effect on the clinical and MRI outcome measures, independently by its impact on lowering the serum cholesterol levels, was the most likely model (Fig. 2 *A* and *B*). The cholesterol-independent model showed a better overall fit than the cholesterol-mediated model. Bootstrapped fit measures for the cholesterol-independent model were the following: CFI = 0.95 (95% CI = 0.86, 1), SRMR = 0.049 (95% CI = 0.02, 0.07), RMSEA = 0.11 (90% CI = 0, 0.18), AIC = 1800 (95% CI = 1,719, 1,892), BIC = 1860 (95% CI = 1,779, 1,952), Akaike weight = 0.71, Schwarz weight = 0.46 (Fig. 2*C*). A direct comparison by computing Akaike weights (Model B Akaike weight/Model A Akaike weight = 0.976/0.023) and Schwarz weights (Model B Schwarz weight/Model A Schwarz weight = 0.704/0.295) suggested that the cholesterol-independent model was considerably more likely than the cholesterol-mediated model (42.24/2.38 times, respectively).

Within the cholesterol-independent model, simvastatin had a significant direct effect on the EDSS (*β* = −0.086, SE = 0.044, *P* = 0.047), a direct effect on brain atrophy (*β* = 0.234, SE = 0.099, *P* = 0.019), and a direct effect on serum cholesterol levels (*β* = −0.739, SE = 0.076, *P* < 0.001). Other model parameters are shown in Fig. 2*A*. Annualized changes in the selected variables are shown in *SI Appendix*, Fig. S3.

### The Bayesian Analysis: Simvastatin Effects on Clinical Outcomes Are Independent of Cholesterol and Are Partially Mediated by Brain Atrophy.

When we calculated how much of the treatment effect was mediated by intermediate variables involved in the pathways of the models discussed above, simvastatin effects on brain atrophy and disability were confirmed to be independent of cholesterol. In particular, simvastatin delayed atrophy directly [treatment → atrophy, *β* = 0.32; 95% CI = 0.09, 0.54], without the mediation of cholesterol (treatment → cholesterol → atrophy, *β* = −0.08; 95% CI = −0.23, 0.07; Fig. 2*B*). Since the 95% CIs of these two parameters do not overlap, the lack of statistical significance for cholesterol-mediated slowing of atrophy is unlikely to be due to the lack of statistical power (Fig. 2*B*).

Similarly, simvastatin directly delayed disability progression, as measured by the EDSS (treatment → EDSS, *β* = −0.139; 95% CI = −0.255, −0.025) without any significant mediation from cholesterol (treatment → cholesterol → EDSS, *β* = 0.014; 95% CI = −0.062, 0.093). Since the 95% CIs of the direct and indirect effects only slightly overlap, this shows that simvastatin effects on EDSS are at least partly independent of cholesterol reduction.

When we investigated the possible mediation of brain atrophy, we found that brain atrophy significantly mediated 31% of the total treatment effect on the EDSS (treatment → atrophy → EDSS, *β* = −0.037; 95% CI = −0.075, −0.010; Fig. 2*B*) and 35% of the total treatment effect on block design score (treatment → atrophy → block design, *β* = 0.33; 95% CI = 0.06, 0.72).

### The Effect of Simvastatin on Brain Atrophy Was Predominant on the Lateral Ventricles and Transverse Temporal Gyrus.

**Fig. 3.**
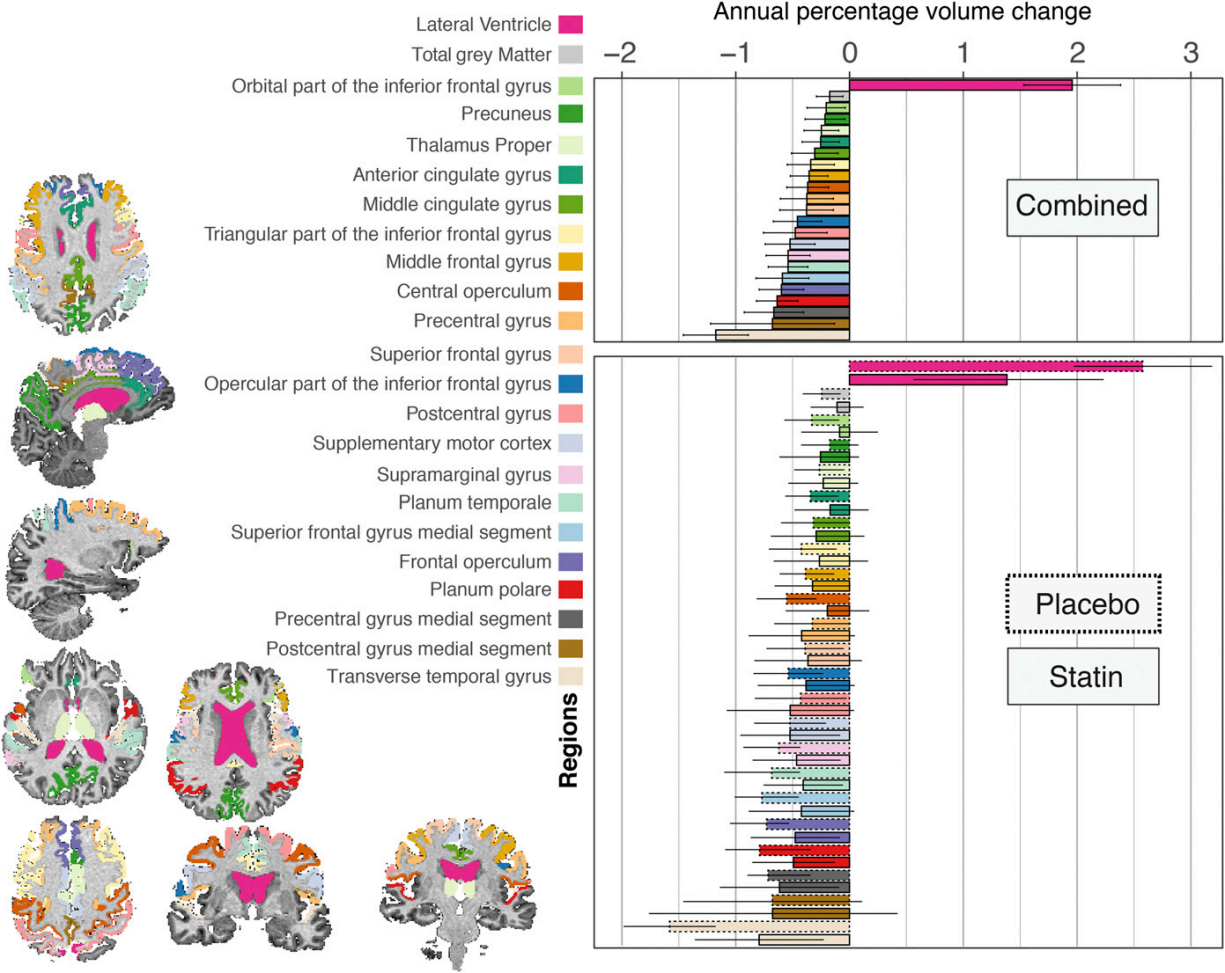
This graph shows the adjusted annual rates of volume loss (or expansion for the lateral ventricles), which are calculated from the coefficient of the interaction of time and treatment group in the mixed-effects models constructed separately for each region. Only regions with significant volume change in the combined placebo and treatment analysis are shown (adjusted for multiple comparisons with the false-discovery method). Different colors correspond to different regions that are shown with the same appearance in *Left* on the T1-weighted scan of one of the patients (chosen at random) and, in the *Right*, as bar plots. Two bar plots are shown; the above shows the rate of change in the combined analysis of placebo and treatment groups on the horizontal axis and different regions on the vertical axis. The lower bar plot shows the rate of change for the same areas for placebo and simvastatin groups separately. This bar plot shows that only the transverse temporal gyrus shows a significant difference in the rate of change when comparing simvastatin and placebo groups. The error bars indicate 95% confidence interval of the rate of change.

In the analysis of the merged treatment and placebo groups several regions showed significant rate of change over time (after adjustment for multiple comparisons), the fastest of which was the lateral ventricles [1.95% annual expansion (95% confidence interval: 1.53%, 2.38%)], followed by the transverse temporal gyrus [estimated annual rate = −1.17% (95% confidence interval: −0.88%, −1.46%)] (Fig. 3). Rates of volume loss in the postcentral and precentral gyri, frontal regions, anterior and middle parts of the cingulate cortex, precuneus, and thalamus were also significant (which implies ongoing volume loss). Fig. 3 shows the full list of regions that showed statistically significant change over time in the merged analysis of treatment and placebo groups.

When comparing placebo and simvastatin groups, the rates of atrophy were numerically slower in several regions in the simvastatin group (Fig. 3), but only the transverse temporal gyrus showed a significantly faster volume loss in placebo than the treated arm [estimated annual rate (95% confidence interval) in placebo group = −1.58% (95% confidence interval: −1.17%, −1.98%); simvastatin group = −0.79% (95% confidence interval: −0.22%, −1.35%)] (*P* = 0.002). The spatial pattern of focal volume loss was similar between the placebo and simvastatin groups on visual inspection and qualitative comparison. There was no significant treatment mediation effect of regional volume loss in the transverse temporal gyrus on EDSS.

## Discussion

We used multivariate structural equation models to explore and test hypothesized causal mechanisms that may explain the observed treatment effect of a potential neuroprotective drug using the simvastatin trial as a model. In this recent phase 2 trial, simvastatin had a direct effect on delaying EDSS worsening and brain atrophy. What mediates this beneficial effect of statin treatment remains unclear as both cholesterol-mediated and cholesterol-independent mechanisms may contribute. In support of the former, various studies have reported that elevated peripheral cholesterol levels are associated with adverse MS outcomes (36, 37). Therefore, it would be reasonable to hypothesize that a reduction in serum cholesterol levels through statin treatment may confer benefit. Our study, however, suggests that these effects were independent of lowered serum cholesterol and, therefore, does not support the hypothesis that simvastatin’s beneficial effects can be attributed to its effects on lowering serum cholesterol levels and its consequent improved hyperlipidemia, which is known to be a comorbidity in MS (3). This does not rule out a pathogenic role for altered lipid metabolism in MS but suggests that key statin-mediated beneficial effector mechanisms may be independent of peripheral cholesterol lowering.

A cholesterol-independent model, therefore, was the most likely option, and mediation models suggested that a reduction in the rate of EDSS worsening was partly (31%) explained by the treatment effects on brain atrophy, and partly (69%) by a separate direct treatment effect. All of these effects were independent of the change in serum cholesterol levels. Our mechanistic approach, also known as mediation analysis, goes beyond correlation analysis and provides causal evidence of association between two variables. This starts by mathematically deconstructing simvastatin effects as cholesterol-mediated or cholesterol-independent and allows an indirect understanding of whether beneficial simvastatin effects are mediated directly via its effect on lowering peripheral cholesterol levels or via other upstream products of the mevalonate pathway (that produces cholesterol). Serum cholesterol is only one of the downstream products of the 3-hydroxy-3-methyl-glutaryl-CoA (HMG-CoA) reductase (part of the mevalonate pathway), an enzyme that is inhibited by simvastatin. Therefore, the independence of treatment effects in MS from the peripheral cholesterol levels does not indicate that the effect is independent of HMG-CoA reductase inhibition and cholesterol synthesis, but points toward a role for intermediate metabolites downstream of HMG-CoA reductase, but upstream of cholesterol. Cholesterol-independent (or pleiotropic) products of this pathway include isoprenoids that prenylate a variety of key signaling proteins that regulate cell function (38) and whose attenuation may have beneficial neuroprotective and vasculoprotective effects. It has been shown in experimental models that simvastatin inhibits brain protein isoprenylation (39).

The central nervous system is highly enriched in cholesterol, especially within myelin, and most of the cholesterol of the nervous system is synthetized de novo and is independent of blood cholesterol (40). Moreover, intermediate substrates of the cholesterol biosynthesis pathway, such as 8,9-unsaturated sterols, could profoundly stimulate myelin formation and repair (41). While the effect of statins on human brain cholesterol levels, which cannot readily be measured in humans, are unclear, experimental animal data suggest that they reduce the de novo synthesis of cholesterol and, consequently, impair remyelination (40, 42), which, in turn, would worsen patient outcomes. Since we have observed positive effects of simvastatin on brain atrophy and disability, it is unlikely that they are due to its possible effect on central cholesterol. Our results suggest that future research should focus on changes in levels of the upstream intermediate metabolites of the cholesterol synthesis pathway, rather than the potential anti-comorbidity effects of statins in progressive MS (43).

It is possible to speculate that statins can reduce brain atrophy and clinical progression through various biological processes that are not linked with peripheral cholesterol level and cholesterol metabolism. For example, statins have effects on leukocyte adhesion through direct stearic interference of the ICAM-1/LFA-1 adhesion molecules (44), can modulate T cell immune response (45), and inhibit CNS leukocyte migration (46). Furthermore, previous work has demonstrated that the benefit of statins in neuroinflammation can be a consequence of their effects on isoprenoid intermediates (independent of cholesterol) in the mevalonate pathways (47). Atorvastatin treatment that caused T cell immune modulation and reversed relapsing and chronic experimental autoimmune encephalomyelitis models, did not affect circulating levels of cholesterol or cholesterol level in the plasma membrane of T cells. Specific isoprenoid intermediates were responsible for immune modulation by atorvastatin, and not molecules within the sterol (cholesterol) synthetic branch downstream of squalene synthase (47). However, our previous report of the MS-STAT trial demonstrated no significant effect of simvastatin on five immunological markers (IFN-γ, IL-4, IL-10, IL-17, and CD4 Fox P3), suggesting that alternative mechanisms such as neuroprotective and vasculoprotective mechanisms could play a role (38, 48).

A strength of our study is the investigation of the spatiotemporal pattern of ongoing atrophy in patients with secondary progressive multiple sclerosis with very long disease duration (21 y). Our regional analysis showed that brain atrophy at the whole-brain level, rather than the regional level, mediated the treatment effect, suggesting that simvastatin has a generalized effect on brain atrophy and does not target a single region (e.g., thalamus) (15). Regional susceptibility of neuroanatomical areas to neurodegeneration manifests by faster percentage of atrophy rates than that of the entire brain. For example, annual percentage volume loss can be up to 4% in the hippocampus in Alzheimer’s disease (49, 50), while it is up to 1% for the entire brain. In MS, the deep gray matter atrophy rates can be up to 1.5% (15), while the whole-brain atrophy is 0.6%. In this study, we found that the highest rate of loss was in the lateral ventricles, which represent a nonspecific, generalized measure of atrophy. Unlike patients with early secondary progressive or primary progressive MS, none of the deep gray matter nuclei showed a higher rate than total brain rate (the thalamic atrophy rate was 0.24%), while the whole-brain volume loss on average was similar to previous studies (0.65%). Similarly, the medulla oblongata volume, which we used as a proxy for spinal cord atrophy (51) (in the absence of spinal cord imaging data), did not show change over time. The slower than expected rate of atrophy in these structures in patients (who had a disease duration of more than 20 y) suggests a floor effect at which the decline of these structures may slow down, while other structures, such as the transverse temporal gyrus, show a faster rate of atrophy in the placebo arm than in the treated group. As we have shown previously (52), patients with longer disease duration have lower rates of atrophy in the spinal cord than patients with shorter disease duration. We can speculate that the transverse temporal gyrus, which is the auditory cortex and responsible for a “basic” function (53), is spared until later stages of secondary progressive MS, which might show a higher rate after exhaustion of other areas. Our results are in line with pathological observations that generalized neurodegeneration may dominate long-standing secondary progressive MS (54–56), while a more selective pattern and ongoing spinal cord atrophy is seen in earlier MS alongside focal inflammation that responds to immunomodulation (54, 57).

A major difference between our study and the previous analyses of MS-STAT (2, 8) is that we calculated rates of change in imaging and clinical outcomes, rather than average differences between treatment groups at each, as previously reported (2, 8). In this study, we performed an independent image analysis and looked at the rate of change, using all three visits (baseline, year 1, and year 2) with mixed-effects models, and found that the rate of change in the block design but not in the frontal assessment battery was significantly different between treated and untreated patients. This is because the frontal assessment battery, unlike the block design, showed a ceiling effect after the first year of the trial, which reduces the rate of change. For this reason, we only included the block design scores in the multivariate mechanistic models. Block design evaluates the visuospatial memory and depends on fine motor coordination (as it is timed) (58). While there was an association between the rate of brain volume loss and the block design test, evidence for an indirect treatment effect on this cognitive outcome was weaker than EDSS. Our results demonstrate that mechanistic multivariate models can quantify and elucidate interrelations of multimodal measures in a clinical trial.

It is important to note that our study is limited by its post hoc nature. While preplanned statistical analyses of clinical trials are the gold standard to compare treatments, post hoc analyses may nevertheless provide information to generate new hypotheses from the wealth of information collected as part of a trial.

In conclusion, we compared mechanistic hypotheses on how a potential neuroprotective drug, simvastatin, can influence imaging, clinical, cognitive, and patient-reported outcomes through changes in peripheral cholesterol level. We found that beneficial effects of simvastatin in secondary progressive MS were independent of circulating cholesterol. Simvastatin affected motor functioning directly, and indirectly by slowing atrophy rates. A weaker simvastatin effect on visuospatial memory was mediated by slowing atrophy rates. Structural equation models can be applied to trials of neurodegenerative disorders to provide potential insight into mechanisms and quantify the pathways underlying disease-worsening and treatment effects.

## Supplementary Material

Supplementary File
