# Supplementary material for: Applying causal models to explore the mechanism of action of simvastatin in progressive multiple sclerosis
Source: Proc Natl Acad Sci U S A. 2019 May 9;116(22):11020–7. doi: 10.1073/pnas.1818978116 (PMC6561162; doi:10.1073/pnas.1818978116)
Supplement: Supplementary File [file pnas.1818978116.sapp.pdf]

Supplemental Material

Supplemental Methods

Image analysis

We performed the following steps (also shown in the **Supplemental Figure 1**):

1. *N4-bias field correction*

We used ANTs software version 2.2 (1) to correct for the scanner-field inhomogeneity in T1 scans. We used Montreal Neurological Institute intracranial mask (2) transferred with diffeomorphic registration (3) to the native space to limit the correction to the cranium.

2. *Symmetric within-subject template construction*

We constructed an isotropic symmetric template per subject using available time-points with iterative rigid registration (4, 5). This step is necessary to avoid bias towards a time-point (e.g., baseline) since it distributes interpolation and segmentation errors across time-points for an unbiased atrophy calculation (6).

3. Symmetric transformation

We transferred T1, PD and T2 scans to the within-subject template by applying the symmetric transformation matrix. We reconstructed scans with B-spline interpolation to minimise blurring artefacts.

4. *Automatic lesion segmentation*

We used Bayesian Model Selection (BaMoS) to segment white matter lesions longitudinally and produce lesion masks(7–9). BaMoS is a multimodal method that integrates PD, T2, and T1 segmentations to provide lesion masks.

5. Manual editing

We used 3D-Slicer (https://www.slicer.org) version 4.6 to manually edit lesion masks acquired from BaMoS.

6. *White matter segmentation*

We used Geodesic Information Flows (GIF)(10) version 3.0 to segment T1 scans and calculate (normal-appearing) white matter masks. This mask enables filling hypointense white matter lesions while avoiding any change in ventricular sizes(11).

7. *T1 hypointense lesion filling*

We used a longitudinal patch-based method to fill hypointense lesions on T1 scans(11). We used white matter mask from the previous step as a reference to fill hypointense lesions. This step minimises erroneous segmentation of hypointense-lesions as grey matter and increases the precision of atrophy estimates as explained elsewhere(11).

8. *Brain segmentation and parcellation*

We used GIF to segment lesion-filled T1 scans into grey matter, white matter, and CSF and to parcellate the brain to ~120 regions according to the Desikan-Killiany-Tourville protocol (http://braincolor.mindboggle.info/index.html)(12). We calculated the volume of each parcellated region by multiplying segmentation probability maps with the voxel volume.

To calculate whole brain percentage atrophy we used SIENA (part of FSL version 5.0)(13). SIENA estimates the rate of atrophy by measuring the shift of brain edge over two separate time-points. To have consistent results between regional and global atrophy that were not limited by the differences in segmentation methods, we used GIF masks within SIENA instead of BET(14) and FAST(15).

Supplemental Methods

We used a linear regression model in which the percentage brain volume change between baseline and two-year follow-up visits was the response variable. This model included treatment allocation as the variable of interest, and the following nuisance variables: age, gender, centre, and EDSS. We calculated treatment effect defined as the adjusted difference between percentage whole brain volume change of the two

treatment groups, divided by the adjusted percentage whole brain volume change in the placebo group. We set the alpha level at 0.05 for all the analyses presented in this work. We adjusted univariate analyses of regional brain volumes for multiple comparisons with the false discovery rate method in R. We used percentage brain volume changes to calculate the effect size (Cohen's d) between placebo and treatment groups and compared it with the original report of this trial that used a different image analysis pipeline (Cohen's d= 0.410) (16).

Univariate analysis of T2 lesion load, clinical and cognitive changes

Since the focus of this study was on dynamic changes, we extended the previous analyses (17) of clinical and cognitive outcomes–which were performed as pairwise average comparisons at each baseline and year two visit–to the analyses of rates of change in the two treatment and placebo groups. We aimed to identify variables with a significant difference in their *rates* of changes between the two groups including all the three visits, and to include them in multivariate mechanistic models (see below). We used univariate linear mixed-effects models in which fixed-effects were time (years from the study entry), and the interaction of time with treatment allocation. Random effects included time nested in "participant". To allow for repeated measures, we included random intercept and slope as correlated random effects. In these models, dependent variables were cognitive or clinical outcomes (seven separate models for T2 lesion load, PASAT, block design, EDSS, Frontal Assessment Battery, and Multiple Sclerosis Impact Scale 29v2 total and its physical subscale). We included age, gender, and centre as extra (nuisance) fixed-effects variables. We used NLME package (18) version 3.1-131 inside R version 3.4.0 (19).

Medulla oblongata volume

In secondary progressive MS the EDSS depends on walking impairment, which is strongly influenced by spinal cord pathology. We therefore extracted the volume of medulla oblongata (details are explained in the Image Analysis section) to test the development of atrophy in this structure, which we used as a proxy for spinal cord atrophy

Supplemental Results

Simvastatin effects on serum cholesterol levels, brain atrophy, and clinical measures: reproduction o results of the MS-STAT trial

Out of 140 randomised, 131 participants completed the trial and were analysed (see **Supplemental Figure 2** for available data at each visit). The baseline characteristics of the participants were given in the main publication of the MS-STAT trial, and summarised in **Supplemental Table 1**. Our longitudinal results confirmed the findings previously described with a new image analysis pipeline and statistical modelling (16). Briefly, there were slower rates of atrophy, EDSS worsening, and worsening of Block Design T-score and the MSIS-29v2 (the physical sub-score) in the treatment arm compared to the placebo group. There was a faster reduction in cholesterol in treatment arm. These results are explained in the Supplemental Material and **Supplemental Figure 3**.

There was a statistically significant difference in the rate of decline of the total cholesterol levels in the simvastatin group compared to the placebo arm (-0.68±0.07 mmol/year *vs.* -0.01±0.05 mmol/year, $p<0.001$) (**Supplemental Figure 3**). The rate of whole brain volume loss over two years was slower in the simvastatin group than in the placebo arm: -0.42 (SD=0.50) vs. -0.657 (SD=0.62), Cohen's *d* or effect size=0.409, $p=0.002$) (**Supplemental Figure 3**). The adjusted difference of the percentage brain volume change between the placebo and active treatment arm was

0.245 (95% confidence interval=0.087 to 0.403). The rate of annual EDSS worsening was slower in the simvastatin group than in the placebo arm (estimated rate ± standard-error 0.08 ± 0.04 vs 0.21 ± 0.03, p=0.002) (**Supplemental Figure 3**). Patients on simvastatin showed a significant difference in the rate of change of block design (0.92 ± 0.45 vs. -0.13 ± 0.33, p=0.04), and on the physical subtest of the Multiple Sclerosis Impact Scale 29v2 (0.26 ± 0.97 ± 0.72 *vs.* 2.37 ± 0.75, p=0.03) compared to patients on placebo (**Supplemental Figure 3**). There were no differences in rates of change between treatment and placebo groups in PASAT (estimated average rate/year ± standard error in placebo: 0.32 ± 0.69 *vs* treatment: 1.47 ± 0.93, p=0.11) and Frontal Assessment Battery (estimated average rate/year ± standard error in placebo: -0.23 ± 0.17 *vs* treatment: 0.28 ± 0.23, p=0.24). There was no change in T2 lesion volume accumulation between patients on simvastatin and those on placebo.

There was no treatment effect on the rate of change in T2 lesion volume

At baseline, lesion volume in the placebo group was 22.14 mL (95%CI: 18.82 to 25.46), which was not different (p=0.33) from the treatment group (average=19.3, 95% CI: 13.48 to 25.12). Lesion volumes significantly increased in each group: average [95%CI] for the treatment group was 0.55 ml/year [0.25 to 0.85], and the average for the placebo group was 0.72 ml/year [0.55 to 0.87]). However, rates of change were similar between treatment and placebo groups.

Medulla oblongata volume

There was no significant change over time in the medulla oblongata volume (average rate of change = -4.83 ml/year, standard error = 10.31, p = 0.64).

Supplemental Discussion

In our candidate models we considered MSIS 29v2 physical sub-score to be the last variable in the sequence. MSIS 29v2 psychological sub-score can theoretically act as an intermediate for the effect of simvastatin on disability and cognitive performance. However, since there was no treatment effect on the psychological sub-score we did not include it in the mechanistic (structural equation) models and considered the physical sub-score as the last in the chain of variables.

We used a novel image analysis pipeline alongside SIENA and reproduced the original findings of the MS-STAT trial independently, which was conducted by boundary-shift integral (BSI) and different segmentation and registration methods. The differences between rates of atrophy between placebo and treatment groups were highly similar agreement (average [95%CI] difference between groups in our study: 0.245 [0.087 to 0.403], and in the original report (16): 0.254 [0.087 to 0.422]). The effect size in this study (0.409) was similar to the original report (0.410), which confirms a small to medium effect of simvastatin on brain atrophy. However, rates of percentage brain volume were slightly higher in our study. For example, this rate for the placebo group was 0.587% annual loss in the original report but it was 0.657% in this study. This is a methodological artefact due to slightly faster average atrophy rates calculated by SIENA (compared to BSI used in the original report). A previous methodological comparison showed that SIENA produces 20% faster atrophy rates, while these two methods had an excellent agreement otherwise (21), which is confirmed by the adjusted difference and similar effect sizes.

Supplemental References

1. Tustison NJ, et al. (2010) N4ITK: improved N3 bias correction. *IEEE Trans Med Imaging* 29(6):1310–1320.

2. Boyes RG, et al. (2008) Intensity non-uniformity correction using N3 on 3-T scanners with multichannel phased array coils. *NeuroImage* 39(4):1752–1762.

3. Avants BB, Epstein CL, Grossman M, Gee JC (2008) Symmetric diffeomorphic image registration with cross-correlation: evaluating automated labeling of elderly and neurodegenerative brain. *Med Image Anal* 12(1):26–41.

4. Reuter M, Fischl B (2011) Avoiding asymmetry-induced bias in longitudinal image processing. *NeuroImage* 57(1):19–21.

5. Leung KK, Ridgway GR, Ourselin S, Fox NC, Alzheimer's Disease Neuroimaging I (2012) Consistent multi-time-point brain atrophy estimation from the boundary shift integral. *Neuroimage* 59(4):3995–4005.

6. Fox NC, Ridgway GR, Schott JM (2011) Algorithms, atrophy and Alzheimer's disease: Cautionary tales for clinical trials. *NeuroImage* 57(1):15–18.

7. Sudre CH, et al. (2015) Bayesian model selection for pathological neuroimaging data applied to white matter lesion segmentation. *IEEE Trans Med Imaging* 34(10):2079–2102.

8. Sudre CH, Cardoso MJ, Ourselin S, Alzheimer's Disease Neuroimaging Initiative (2017) Longitudinal segmentation of age-related white matter hyperintensities. *Med Image Anal* 38:50–64.

9. Carass A, et al. (2017) Longitudinal multiple sclerosis lesion segmentation: Resource and challenge. *NeuroImage* 148:77–102.

10. Cardoso MJ, et al. (2015) Geodesic information flows: spatially-variant graphs and their application to segmentation and fusion. *IEEE Trans Med Imaging* 34(9):1976–1988.

11. Prados F, et al. (2016) A multi-time-point modality-agnostic patch-based method for lesion filling in multiple sclerosis. *NeuroImage* 139:376–384.

12. Klein A, Tourville J (2012) 101 labeled brain images and a consistent human cortical labeling protocol. *Front Neurosci* 6. doi:10.3389/fnins.2012.00171.

13. Smith SM, De Stefano N, Jenkinson M, Matthews PM (2001) Normalized accurate measurement of longitudinal brain change. *J Comput Assist Tomogr* 25(3):466–475.

14. Smith SM (2002) Fast robust automated brain extraction. *Hum Brain Mapp* 17(3):143–55.

15. Zhang Y, Brady M, Smith S (2001) Segmentation of brain MR images through a hidden Markov random field model and the expectation-maximization algorithm. *IEEE Trans Med Imaging* 20(1):45–57.

16. Chataway J, et al. (2014) Effect of high-dose simvastatin on brain atrophy and disability in secondary progressive multiple sclerosis (MS-STAT): a randomised, placebo-controlled, phase 2 trial. *Lancet Lond Engl* 383(9936):2213–2221.

17. Chan D, et al. (2017) Effect of high-dose simvastatin on cognitive, neuropsychiatric, and health-related quality-of-life measures in secondary progressive multiple sclerosis: secondary analyses from the MS-STAT randomised, placebo-controlled trial. *Lancet Neurol* 16(8):591–600.

18. Pinheiro J, Bates D, DebRoy S, Sarkar D, R Core Team (2017) nlme: Linear and Nonlinear Mixed Effects Models. Available at: https://cran.r-project.org/web/packages/nlme/citation.html [Accessed October 18, 2017].

19. R Core Team (2014) *R: A Language and Environment for Statistical Computing* (R Foundation for Statistical Computing, Vienna, Austria) Available at: http://www.R-project.org/.

20. Liptak Z, et al. (2008) Medulla oblongata volume: a biomarker of spinal cord damage and disability in multiple sclerosis. *AJNR Am J Neuroradiol* 29(8):1465–1470.

21. Smith SM, et al. (2007) Longitudinal and cross-sectional analysis of atrophy in Alzheimer's disease: Cross-validation of BSI, SIENA and SIENAX. *NeuroImage* 36(4):1200–1206.

Supplemental Figures

Supplemental Figure 1. Image analysis pipeline

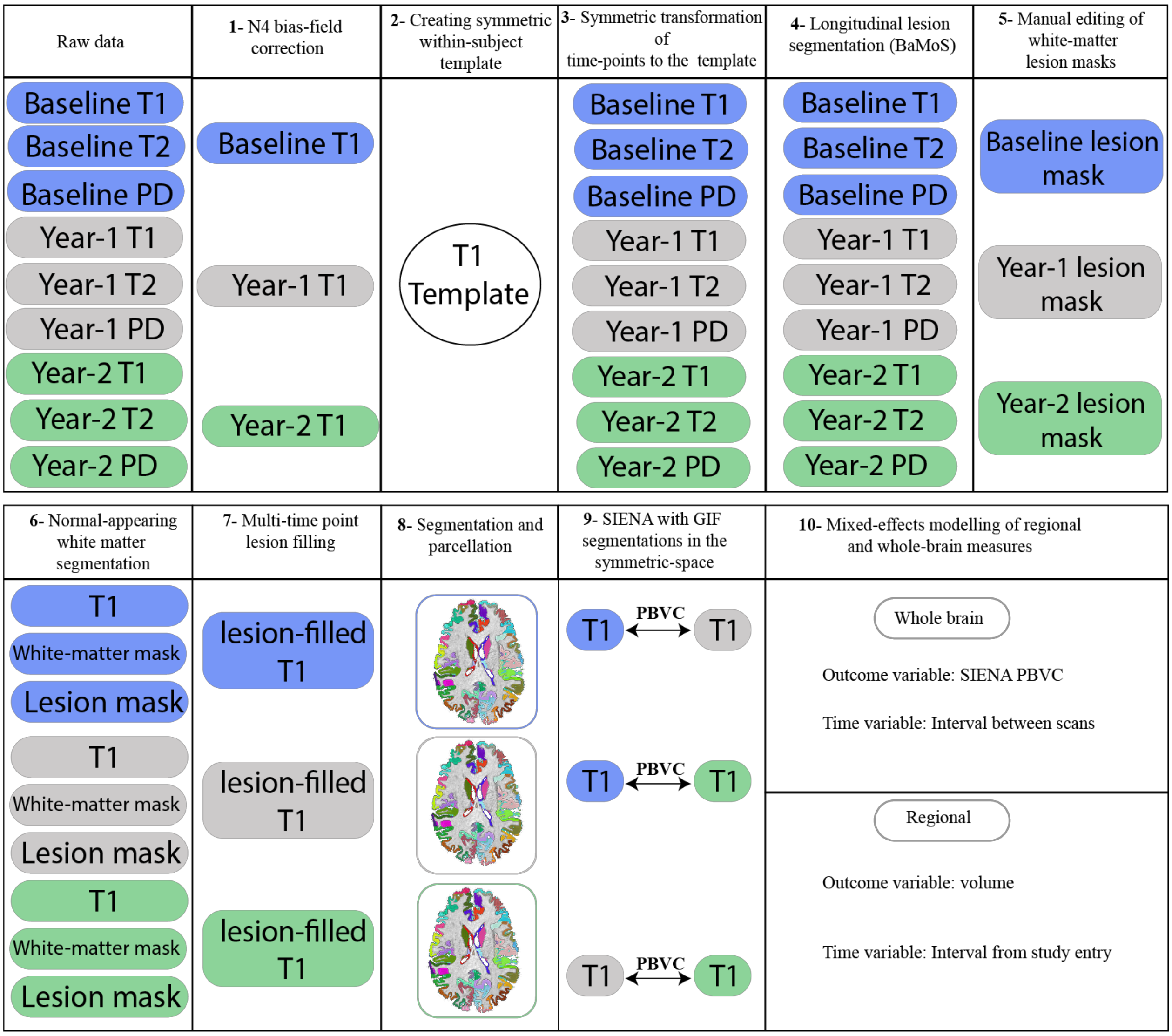

Legend: This diagram shows different steps of a longitudinal image analysis pipeline. This pipeline is designed to provide unbiased atrophy rates.

Supplemental Figure 2. Available trial data.

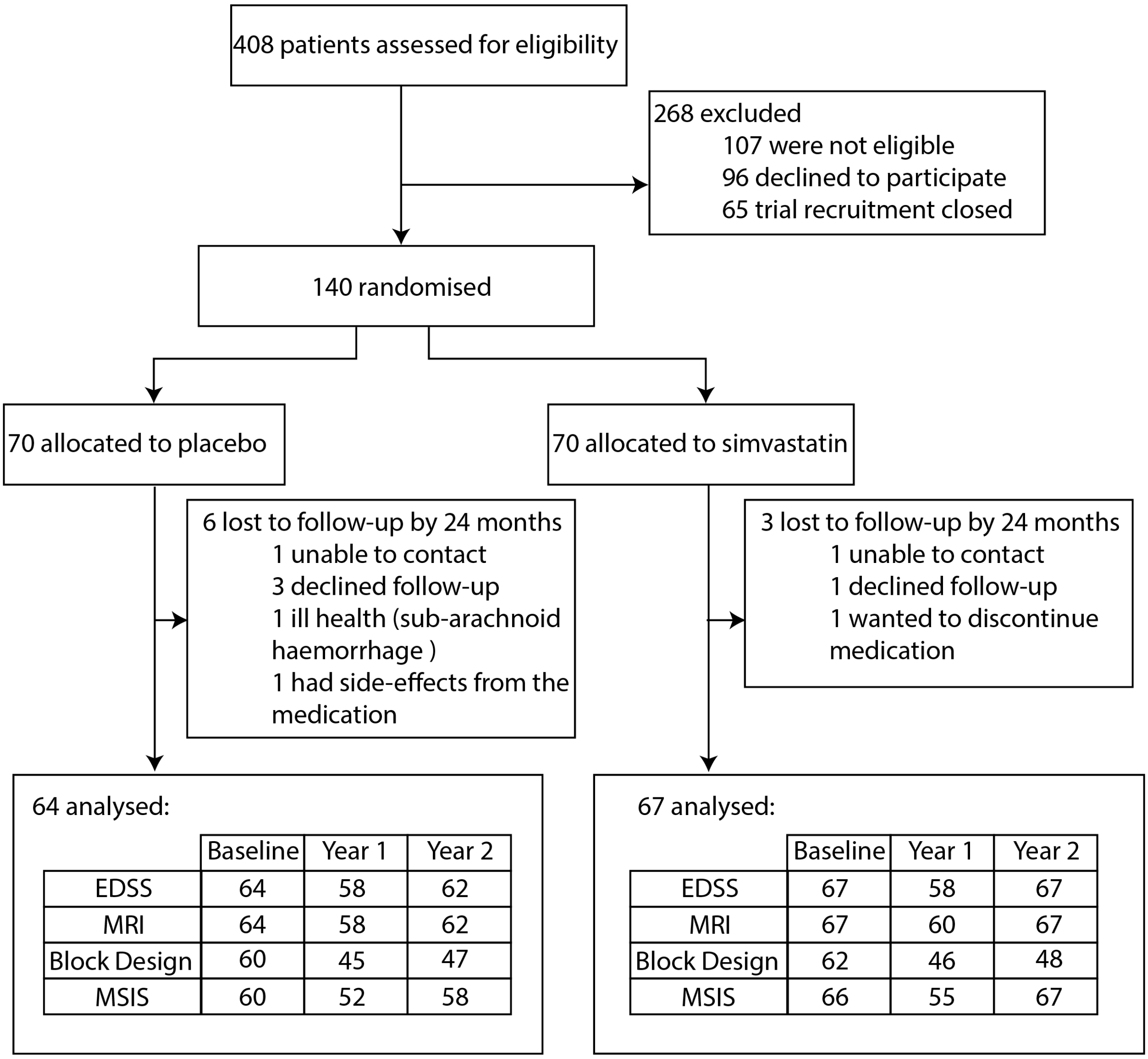

64 analysed:

	Baseline	Year 1	Year 2
EDSS	64	58	62
MRI	64	58	62
Block Design	60	45	47
MSIS	60	52	58

67 analysed:

	Baseline	Year 1	Year 2
EDSS	67	58	67
MRI	67	60	67
Block Design	62	46	48
MSIS	66	55	67

Legend. This diagram shows the flow of participants from screening to inclusion in the MS-STAT trial. Available clinical, cognitive, and imaging variables are shown in the table for all the three visits.

EDSS; Expanded Disability Status Scale, MRI; magnetic resonance imaging, MSIS; Multiple Sclerosis Impact Scale.

Supplemental Figure 3. Annualised change in outcomes with a significant treatment effect.

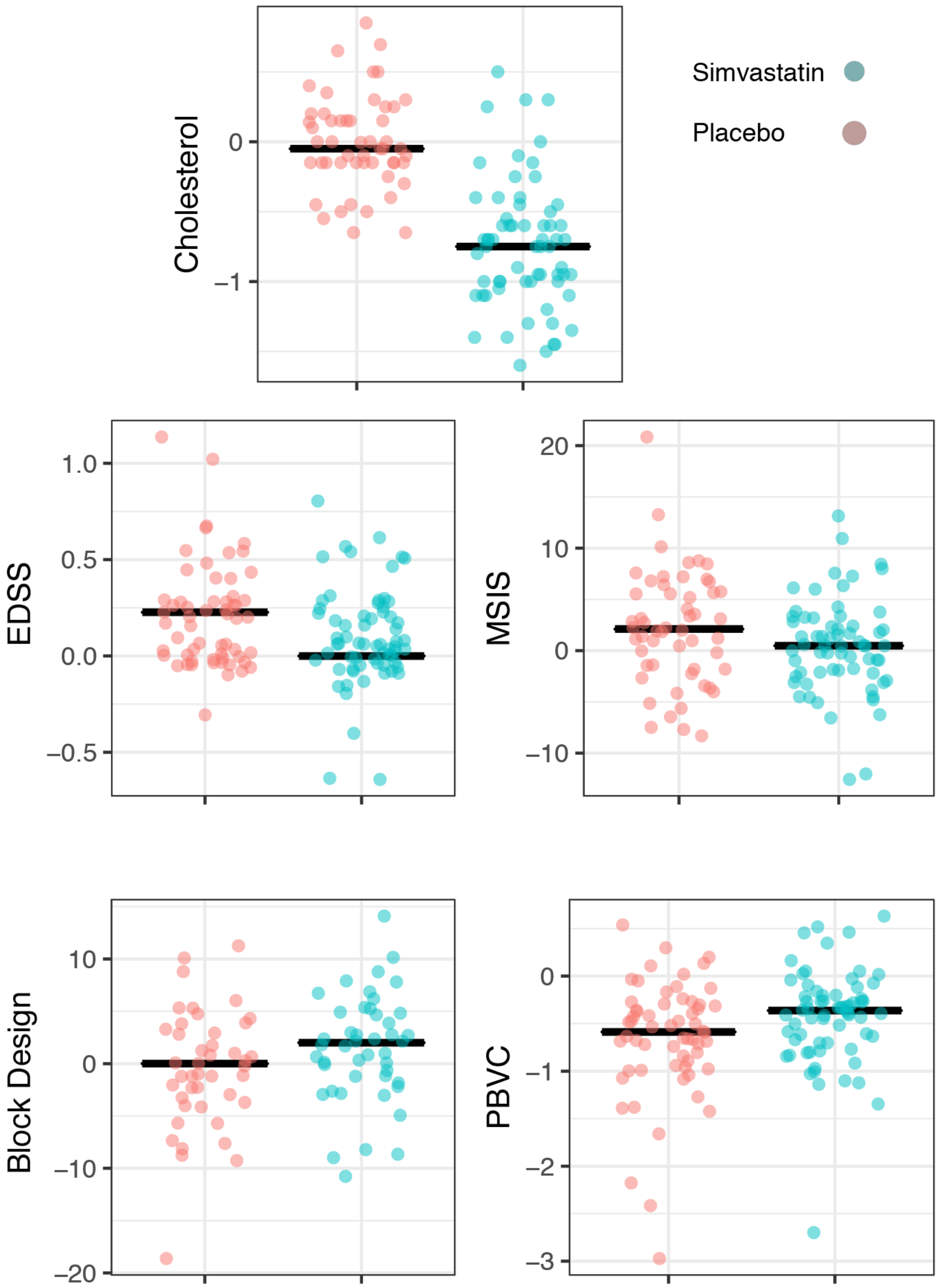

Legend. The annual rate of changes for MRI, clinical, cognitive and patient-reported outcomes included in the mechanistic models (EDSS rates are jittered vertically by 0.1 to enable visualising overlapping values). In each of the four plots, horizontal black lines show the medians of the variable shown on *y*-axes, for placebo (blue) and statin groups (red).

EDSS; Expanded Disability Status Scale, MSIS; Multiple Sclerosis Impact Scale, PBVC; percentage brain volume change.

Supplemental Table 1. Baseline characteristics of the participants.

	**Treatment**	**Placebo**	**Combined**
No. of participants	67	64	131
Average age in years (SD)	51.58 (7.03)	50.9 (6.94)	51.2 (6.97)
Females (males)	46 (21)	42 (22)	88 (43)
Duration of SPMS in years (SD)	7.41 (5.74)	6.87 (4.62)	7.14 (5.21)
Duration of MS in years (SD)	22.32 (8.27)	19.97 (8.94)	21.17 (8.65)
Median EDSS (range)	6 (6-6.5)	6 (4-7)	6 (4-7)
Average years of education (SD)	13.69 (3.07)	13.42 (3.2)	13.56 (3.16)

Abbreviations: SD; standard deviation, SPMS; secondary-progressive multiple sclerosis, MS; multiple sclerosis, EDSS; Kurtzke's Expanded-Disability Status Scale.
